# New Insights into Hepatic and Intestinal Microcirculation and Pulmonary Inflammation in a Model of Septic Shock and Veno-Arterial Extracorporeal Membrane Oxygenation in the Rat

**DOI:** 10.3390/ijms25137421

**Published:** 2024-07-06

**Authors:** Fabian Edinger, Lena Holtz, Götz Schmidt, Emmanuel Schneck, Thomas Zajonz, Michael Sander, Christian Koch

**Affiliations:** Department of Anesthesiology, Critical Care Medicine and Pain Therapy, University Hospital of Giessen, Justus-Liebig-University, 35392 Giessen, Germanychristian.koch@chiru.med.uni-giessen.de (C.K.)

**Keywords:** V-A ECMO, inflammation, sepsis, intestinal perfusion

## Abstract

Despite significant efforts toward improving therapy for septic shock, mortality remains high. Applying veno-arterial (V-A) extracorporeal membrane oxygenation (ECMO) in this context remains controversial. Since the cannulation of the femoral artery for V-A ECMO return leads to lower body hyperoxia, this study investigated the impact of V-A ECMO therapy on the intestinal and hepatic microcirculation during septic shock in a rodent model. Thirty male Lewis rats were randomly assigned to receive V-A ECMO therapy with low (60 mL/kg/min) or high (90 mL/kg/min) blood flow or a sham procedure. Hemodynamic data were collected through a pressure-volume catheter in the left ventricle and a catheter in the lateral tail artery. Septic shock was induced by intravenous administration of lipopolysaccharide (1 mg/kg). The rats received lung-protective ventilation during V-A ECMO therapy. The hepatic and intestinal microcirculation was measured by micro-lightguide spectrophotometry after median laparotomy for two hours. Systemic and pulmonary inflammation was detected via enzyme-linked immunosorbent assays (ELISA) of the plasma and bronchoalveolar lavage (BAL), respectively, measuring tumor necrosis factor-alpha (TNF-α), interleukins 6 (IL-6) and 10 (IL-10), and C-X-C motif ligands 2 (CXCL2) and 5 (CXCL5). Oxygen saturation and relative hemoglobin concentration were reduced in the hepatic and intestinal microcirculation during V-A ECMO therapy, independent of the blood flow rate. Further, rats treated with V-A ECMO therapy also presented elevated systolic, diastolic, and mean arterial blood pressure and increased stroke volume, cardiac output, and left ventricular end-diastolic volume. However, left ventricular end-diastolic pressure was only elevated during high-flow V-A ECMO therapy. Blood gas analysis revealed a dilutional anemia during V-A ECMO therapy. ELISA analysis showed an elevated plasma CXCL2 concentration only during high-flow V-A ECMO therapy and elevated BAL CXCL2 and CXCL5 concentrations only during low-flow V-A ECMO therapy. Rats undergoing V-A ECMO therapy exhibited impaired microcirculation of the intestine and liver during septic shock despite increased blood pressure and cardiac output. Increased pulmonary inflammation was detected only during low-flow V-A ECMO therapy in septic shock.

## 1. Introduction

Despite numerous efforts to improve the therapy for sepsis and septic shock, mortality rates remain high [1]. The international Surviving Sepsis Campaign defines sepsis as a dysregulated host response to infection resulting in life-threatening organ dysfunction [2]. Septic shock is defined as sepsis with persisting hypotension and elevated serum lactate concentrations >2 mmol/L despite adequate volume resuscitation [2]. Cytokine release and endothelial damage are associated with reduced peripheral vascular resistance, resulting in vasoplegia [3]. Common treatment options include administering norepinephrine and vasopressin [4]. However, current guidelines recommend using dobutamine or epinephrin to treat cardiac dysfunction with persistent hypoperfusion [4]. While there is no consensus on the definition of septic cardiomyopathy, many define it as acute uni- or bi-ventricular systolic or diastolic dysfunction with reduced contractility not due to coronary disease [5]. The incidence of septic cardiomyopathy varies widely from 10% to 70% [6].

While venovenous (V-V) extracorporeal membrane oxygenation (ECMO) has gained wide acceptance during the last two decades due to supporting evidence from the CESAR trial and the clinical experiences through the two large respiratory virus pandemics (influenza in 2009 and severe acute respiratory syndrome coronavirus 2 in 2020), veno-arterial (V-A) ECMO has mainly been restricted to cardiac surgery for many years [7,8,9]. With the incremental peripheral cannulation of the femoral artery for ECMO return, V-A ECMO has been increasingly applied outside cardiac surgery over the last decade. Indications for peripheral V-A ECMO therapy include cardiogenic shock or circulatory arrest.

Applying V-A ECMO during septic shock remains controversial. While a retrospective, multicenter, international cohort study indicated a survival rate of 60% for patients with sepsis-induced cardiomyopathy treated with V-A ECMO, other studies have reported mortality rates of 63.6% and 76.7% regardless of cardiac function [10,11]. The large foreign surface of the ECMO circuit and membrane is associated with endothelial damage and activation of the complement, inflammatory, and coagulatory systems, known as ECMO-induced inflammation [12]. The ECMO membrane can also be colonized by circulating bacteria [13,14]. During sepsis, loss of the tight junctions between endothelial cells is associated with an increased capillary permeability and reduced perfusion of tissues [15]. In the gut, this phenomenon can be associated with the translocation of bacteria into the blood, leading the gut to be called the “motor of sepsis” [16].

It must be underlined that the femoral cannulation of V-A ECMO leads to a retrograde blood flow, resulting in a mixing zone of cardiac output (CO) and ECMO inflow [17,18]. Therefore, the CO is influenced by the inspiratory oxygen fraction (F_i_O_2_) of the ventilation [19]. However, since measuring the intestinal microcirculation is challenging in critically ill patients, the impact of the retrograde injection of highly oxygenated blood on the intestinal barrier is unknown.

Therefore, the primary aim of this study was to investigate the intestinal microcirculation during septic shock treated with femoral V-A ECMO therapy in a rat model. Its secondary aim was to evaluate the inflammatory response in the lung during sepsis in rats undergoing V-A ECMO therapy.

## 2. Results

### 2.1. Intestinal and Hepatic Microcirculation

**Figure 1:**
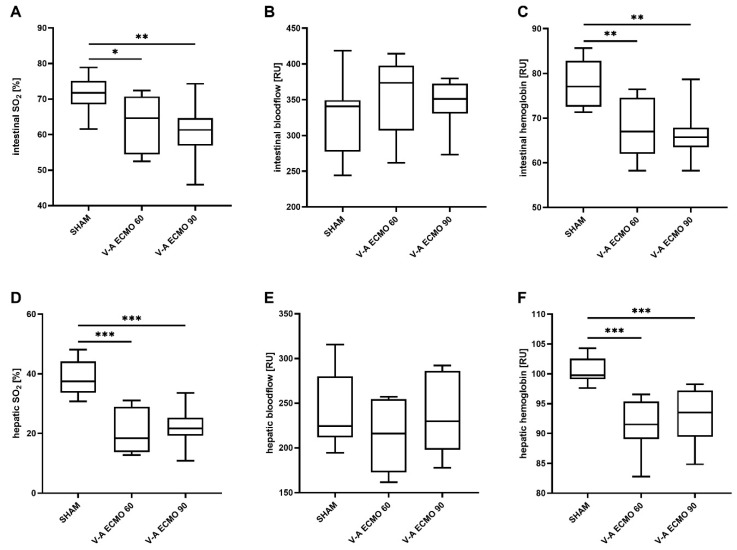
Means of the time course of intestinal (**A**) regional oxygen saturation, (**B**) relative blood flow, (**C**) relative hemoglobin concentration, hepatic (**D**) regional oxygen saturation, (**E**) relative blood flow, and (**F**) relative hemoglobin concentration. Rats treated with V-A ECMO showed reduced regional oxygen saturation and relative hemoglobin of the intestine and liver compared to sham animals. Relative blood flow in the intestine and liver did not differ significantly between V-A ECMO therapy and sham procedure. The asterisks denote the degree of statistical significance: *, *p* < 0.05; **, *p* < 0.01; ***, *p* < 0.001. Abbreviations: ECMO = extracorporeal membrane oxygenation; RU = relative units; SO_2_ = tissue oxygen saturation; V-A = veno-arterial.

Intestinal tissue oxygenation saturation (SO_2_) and relative hemoglobin concentrations measured by white light and laser Doppler spectrometry were lower during V-A ECMO therapy with low (60 mL/kg/min; V-A ECMO 60) and high (90 mL/kg/min; VA-ECMO 90) blood flow than during a sham procedure (SO_2_: sham vs. V-A ECMO 60, *p* = 0.042; sham vs. V-A ECMO 90, *p* = 0.006; relative hemoglobin: sham vs. V-A ECMO 60, *p* = 0.003; sham vs. V-A ECMO 90, *p* = 0.001; Figure 1A,C). Additionally, hepatic SO_2_ and relative hemoglobin concentrations were lower during high- and low-flow V-A ECMO therapy than sham therapy (all *p* < 0.001; Figure 1D,F). However, intestinal and hepatic blood flow did not differ significantly between high- and low-flow V-A ECMO therapy and sham animals (intestine: sham vs. V-A ECMO 60, *p* = 0.306; sham vs. V-A ECMO 90, *p* = 0.706; liver: sham vs. V-A ECMO 60, *p* = 0.388; sham vs. V-A ECMO 90, *p* = 1.000; Figure 1B,E).

### 2.2. Hemodynamic Parameters

**Figure 2:**
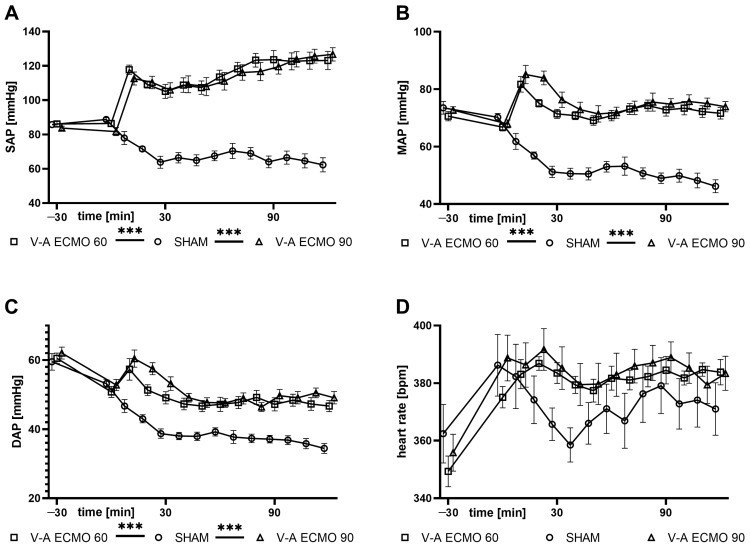
Time course of (**A**) SAP, (**B**) MAP, (**C**) DAP, and (**D**) heart rate. SAP, DAP, and MAP were measured elevated during low- and high-flow V-A ECMO therapy and septic shock than during sham procedure. Heart rate did not differ significantly between low- and high-flow V-A ECMO therapy and sham therapy. The asterisks denote the degree of statistical significance: ***, *p* < 0.001. Abbreviations: DAP = diastolic arterial pressure; ECMO = extracorporeal membrane oxygenation; MAP = mean arterial pressure; SAP = systolic arterial pressure; V-A = veno-arterial.

Systolic (SAP), diastolic (DAP), and mean (MAP) arterial blood pressures were higher with low- and high-flow V-A ECMO therapy compared to the sham procedure (all *p* < 0.001; Figure 2). However, heart rate did not differ significantly between low- and high-flow V-A ECMO therapy and sham therapy (sham vs. V-A ECMO 60: *p* = 0.562; sham vs. V-A ECMO 90: *p* = 0.358; Figure 2).

**Figure 3:**
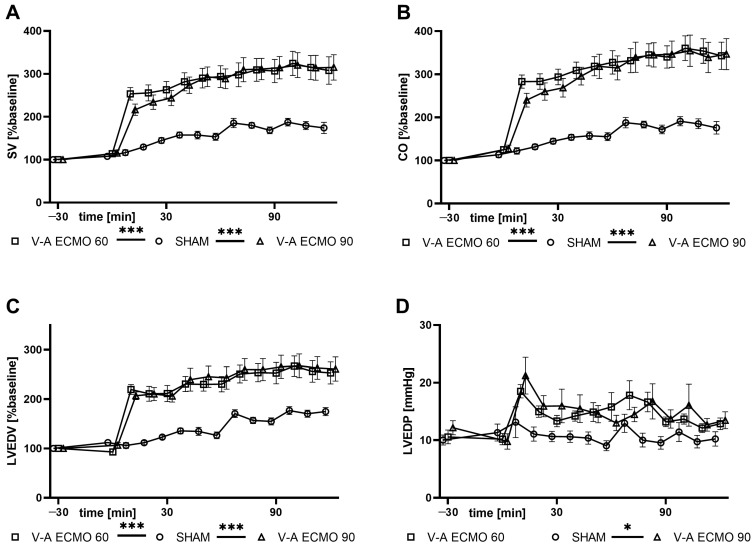
Time course of (**A**) SV, (**B**) CO, (**C**) LVEDV, and (**D**) LVEDP. While SV, CO, and LVEDV were significantly higher with low- and high-flow V-A ECMO therapy than sham procedure, LVEDP was only significantly higher with high-flow V-A ECMO therapy than sham procedure. The asterisks denote the degree of statistical significance: *, *p* < 0.05; ***, *p* < 0.001. Abbreviations: CO = cardiac output; ECMO = extracorporeal membrane oxygenation; LVEDP = left ventricular end-diastolic pressure; LVEDV = left ventricular end-diastolic volume; SV = stroke volume.

Analysis of the pressure-volume data revealed that stroke volume (SV), CO, and left ventricular end-diastolic volume (LVEDV) were higher with low- and high-flow V-A ECMO compared to the sham group (all *p* < 0.001; Figure 3). Moreover, left ventricular end-diastolic pressure (LVEDP) was significantly elevated during high-flow (*p* = 0.039) and marginally higher during low-flow (*p* = 0.069) V-A ECMO therapy compared to the sham group (Figure 3). However, left ventricular ejection fraction (LVEF) did not differ significantly between low- and high-flow V-A ECMO therapy and sham procedure (sham vs. V-A ECMO 60: *p* = 0.512; sham vs. V-A ECMO 90: *p* = 1.000; Figure S1).

### 2.3. Blood Gas Analysis

While arterial oxygen saturation (S_a_O_2_) and arterial partial pressure of oxygen (pO_2_) were measured as significantly elevated during high- and low-flow V-A ECMO therapy compared to the sham group (all *p* < 0.001), central venous oxygen saturation (S_cv_O_2_) did not differ significantly between the groups (V-A ECMO 60: *p* = 0.675; V-A ECMO 90: *p* = 0.966; Table 1). Furthermore, analysis of the arterio-venous oxygen difference revealed no differences between the groups (V-A ECMO 60: *p* = 0.364; V-A ECMO 90: *p* = 1.000; Table 1). Additionally, arterial partial pressure of carbon dioxide (pCO_2_) was significantly elevated in sham animals than during low- and high-flow V-A ECMO therapy, with consequent reductions in pH and base excess (all *p* < 0.001; Table 1). Moreover, while hemoglobin and hematocrit concentrations were measured as decreased during high- and low-flow V-A ECMO therapy compared to the sham group (all *p* < 0.001), lactate concentrations only trended towards higher values during high-flow V-A ECMO therapy (V-A ECMO 60: *p* = 1.000; V-A ECMO 90: *p* = 0.066; Table 1). Furthermore, there were elevated concentrations of chloride during low- and high-flow V-A ECMO therapy compared to the sham procedure (V-A ECMO 60: *p* < 0.001; V-A ECMO 90: *p* = 0.001; Table 1). However, sodium (V-A ECMO 60: *p* = 0.411; V-A ECMO 90: *p* = 0.411), potassium (V-A ECMO 60: *p* = 1.0; V-A ECMO 90: *p* = 0.905), and calcium (V-A ECMO 60: *p* = 0.097; V-A ECMO 90: *p* = 0.227) did not differ significantly between low- and high-flow V-A ECMO therapy and sham procedure, while glucose concentrations trended towards higher values during sham procedure than low- and high-flow V-A ECMO therapy (V-A ECMO 60: *p* = 0.097; V-A ECMO 90: *p* = 0.050; Table 1

### 2.4. Inflammatory Parameters

**Figure 4:**
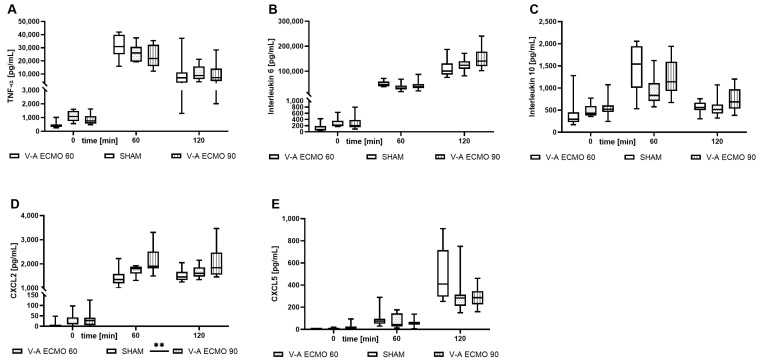
Time course of the plasma concentrations of the inflammatory parameters (**A**) TNF-α, (**B**) IL-6, (**C**) IL-10, (**D**) CXCL2, and (**E**) CXCL5. While CXCL2 concentrations were significantly higher with high-flow V-A ECMO therapy than sham procedure, TNF-α, IL-6, IL-10, and CXCL5 concentrations did not differ significantly between low- and high-flow V-A ECMO therapy and sham animals. The asterisks denote the degree of statistical significance: **, *p* < 0.010. Box and whisker plots indicate the median, interquartile range (box), and minimum and maximum (whiskers). Abbreviations: CXCL2 = C-X-C motif ligand 2; CXCL5 = C-X-C motif ligand 5; TNF-α = tumor necrosis factor alpha.

While increased concentrations of C-X-C motif ligand 2 (CXCL2) were measured during high-flow V-A ECMO therapy (*p* = 0.005), no differences were seen regarding low-flow V-A ECMO therapy and sham procedure (*p* = 0.791). However, tumor necrosis factor-alpha (TNF-α; V-A ECMO 60: *p* = 1.000; V-A ECMO 90: *p* = 0.937), interleukin 6 (IL-6; V-A ECMO 60: *p* = 1.000; V-A ECMO 90: *p* = 0.515), interleukin 10 (IL-10; V-A ECMO 60: *p* = 0.110; V-A ECMO 90: *p* = 1.000), and C-X-C motif ligand 5 (CXCL5; V-A ECMO 60: *p* = 0.189; V-A ECMO 90: *p* = 0.089) concentrations did not differ significantly between low- and high-flow therapy and sham procedure (Figure 4).

**Figure 5:**
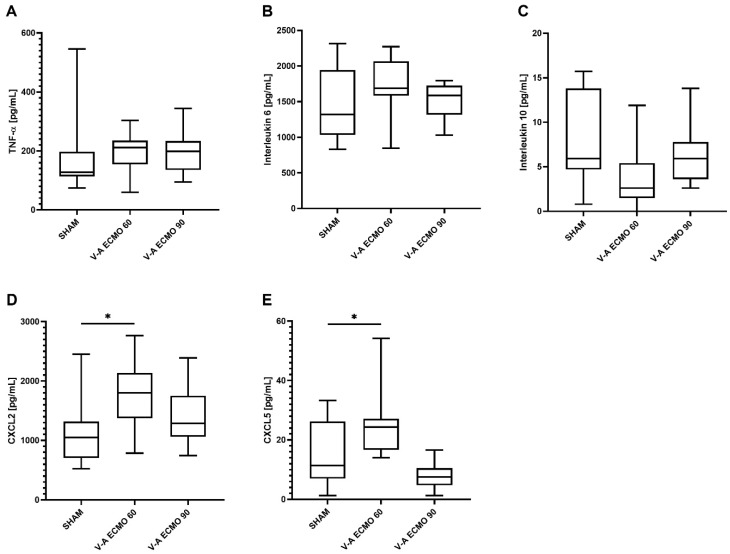
The concentrations of the inflammatory (**A**) TNF-α, (**B**) IL-6, (**C**) IL-10, (**D**) CXCL2, and (**E**) CXCL5 in the BAL. While CXCL2 and CXCL5 concentrations were significantly higher during low-flow V-A ECMO than sham therapy, TNF-α, IL-6, and IL-10 concentrations did not differ significantly between low- and high-flow V-A ECMO therapy and sham procedure. The asterisks denote the degree of statistical significance: *, *p* < 0.05. Box and whisker plots indicate the median, interquartile range (box), and minimum and maximum (whiskers). Abbreviations: CXCL2 = C-X-C motif ligand 2; CXCL5 = C-X-C motif ligand 5; TNF-α = tumor necrosis factor alpha.

While elevated concentrations of CXCL2 (*p* = 0.038) and CXCL5 (*p* = 0.040) were captured in bronchoalveolar lavage (BAL) during low-flow V-A ECMO, no differences were captured in high-flow V-A ECMO animals (CXCL2: *p* = 0.810; CXCL5: p = 0.472; Figure 5). Moreover, the BAL concentrations of TNF-α (V-A ECMO 60: *p* = 1.000; V-A ECMO 90: *p* = 1.000), IL-6 (V-A ECMO 60: *p* = 0.457; V-A ECMO 90: *p* = 1.000), and IL-10 (V-A ECMO 60: *p* = 0.070; V-A ECMO 90: *p* = 0.991) did not differ significantly between low- and high-flow V-A ECMO therapy and sham procedure (Figure 5).

## 3. Discussion

To the best of our knowledge, this is the first description of a live model of intestinal microcirculation during V-A ECMO therapy with septic shock in the rat. Briefly, this study demonstrated reduced SO_2_ and relative hemoglobin concentration in the intestine and liver during septic shock and V-A ECMO therapy. Additionally, rats treated with V-A ECMO presented elevated SAP, DAP, and MAP and increased SV, CO, and LVEDV. LVEDP was only elevated during high-flow V-A ECMO therapy. Moreover, cytokine analysis revealed elevated serum CXCL2 concentrations during high-flow V-A ECMO therapy. Furthermore, BAL analysis showed elevated CXCL2 and CXCL5 concentrations only during low-flow V-A ECMO therapy.

Due to its lower risk profile than central access with sternotomy, femoral cannulation is frequently used for V-A ECMO therapy. Therefore, the topic of this study is of great interest. It has been shown that the retrograde ECMO blood flow through the femoral artery results in a mixing zone with the native CO [17,18]. Consequently, the lower body is at high risk for hyperoxia during V-A ECMO therapy. Therefore, increased SO_2_ in the intestine and liver were expected during V-A ECMO therapy. However, our study found decreased SO_2_ in the intestine and liver.

Given the lack of comparable studies on V-A ECMO therapy during septic shock, we compare our results to those of humans during cardiopulmonary bypass (CPB) and animal models of septic shock. Consistent with our results, Nahum et al. found reduced hepatic oxygen saturation with near-infrared spectroscopy during lipopolysaccharide (LPS)-induced septic shock in piglets [20]. Additionally, Albuszies et al. reported an increased hepatic and maintained intestinal oxygen saturation measured with laser Doppler flowmetry in a mouse model of septic shock [21]. Because septic shock was induced by cecal ligation and the mice received a continuous infusion of hydroxyethyl starch and norepinephrine, these results cannot be directly compared with our study [21].

Inconsistent with our results, jejunal mucosal perfusion was found to be increased with laser Doppler flowmetry in humans during and one hour after CPB [22]. Unlike the femoral cannulation of the V-A ECMO in our study, the heart–lung machine used during CPB is cannulated centrally [22]. Additionally, that study examined humans without septic shock. Therefore, these results cannot be compared with our study.

Since no differences were seen between the low- and high-flow ECMO groups, blood flow seems not to impact the reduction in intestinal and hepatic SO_2_. Interestingly, our previous studies with septic rats treated with V-V ECMO showed the same results regarding hepatic and intestinal microcirculation. Based on this, a systemic effect by the ECMO circuit rather than the cannulation site could be hypothesized.

Analysis of the macrocirculation revealed a progressive decrease in blood pressure in the sham group, which can be explained by the vasodilatation effect of LPS. Interestingly, low- and high-flow V-A ECMO therapy increased the SAP, DAP, and MAP. Since the oxygenated blood from the ECMO is being pumped into an artery, these results were expected and are consistent with our previous studies on V-A ECMO therapy in rats [19,23,24]. However, no differences regarding heart rate were observed between groups, consistent with our previous studies on V-A ECMO therapy in rats [19,23,24]. Interestingly, rats treated with LPS and V-V ECMO showed an increased cardiac rate. Based on the results of this study, this difference could be explained by the different cannulation sites.

Analysis of the pressure-volume catheter data revealed increased SV and CO during V-A ECMO therapy regardless of the blood flow. These results are also consistent with our previous findings in healthy rats [19,23,24]. Interestingly, no differences were seen between the low- and high-flow V-A ECMO groups, underscoring that CO was also increased during low blood flow. It should be noted that the pressure-volume catheter is inserted into the left ventricle through the aortic valve resulting in an aortic valve insufficiency. Therefore, the increased LVEDV could be caused by the femoral return of the oxygenated blood. Since the heart is unloaded during V-A ECMO therapy due to jugular drainage, the returning V-A ECMO blood appears to be ejected during the systole, resulting in an increased stroke volume and cardiac output. While LVEDV was elevated during both low- and high-flow V-A ECMO therapy, LVEDP was increased only during high-flow V-A ECMO therapy. Therefore, the effect of the increased afterload from femoral cannulation on the LVEDP may be correlated with blood flow. Unloading of the heart during V-A ECMO therapy potentially explains why no differences in LVEF were seen among groups, although LVEDV was elevated during V-A ECMO therapy. Nevertheless, the mixing zone may be influenced by cardiac output and may be located distally during low-flow V-A ECMO therapy. Because pulmonary function is impaired by lipopolysaccharide infusion, animals are at risk for cardiac and intestinal ischemia. However, data from veno-venous ECMO studies during septic shock in rats with high blood flow also showed reduced intestinal microcirculation. Therefore, the intestinal microcirculation seems to be influenced more by systemic effects. Nevertheless, impaired intestinal oxygenation due to cardiac output of decreased oxygenated blood cannot be excluded.

While increased blood pressure and CO as well as decreased hepatic and intestinal SO_2_ and relative hemoglobin concentrations were seen during V-A ECMO therapy, further analyses focused on the blood gases and hemoglobin concentrations. S_a_O_2_ and pO_2_ were increased during V-A ECMO therapy despite lung protective ventilation and independent of blood flow, highlighting that this model works well. Rats in the sham group exhibited respiratory acidosis with increased pCO_2_ due to impaired pulmonary function. These alterations could be caused by the cytokine storm induced by the LPS infusion [25].

The hyperchloridemia observed during ECMO therapy may reflect the priming of the ECMO circuit with unbalanced hydroxy ethyl starch containing 154 mmol/L of sodium and chloride. Rats in the V-A ECMO groups presented dilutional anemia. Furthermore, because of the macrohematuria seen in some animals at the end of the experiments, hemolysis could also contribute to the anemia. Since no increase in lactate concentrations was observed, the critical hemoglobin concentration was not reached. Nevertheless, the reduced hemoglobin concentration may explain the reduced hepatic and intestinal SO_2_. Since the hematocrit influences blood viscosity, the dilutional anemia may have improved the rheology of the blood. The best oxygenation of the rat liver was found at a hematocrit of 20% [26]. While even higher hematocrit values were recorded during our study, an increased hepatic and intestinal microcirculation should have been expected with V-A ECMO therapy compared to sham procedure without hemodilution. Consistent with this assumption, Thorén et al. found increased jejunal mucosal perfusion during CPB with hemodilution assessed by endoluminal laser Doppler flowmetry in humans [27]. However, they examined CPB in humans without septic shock. Following this, it could be hypothesized that the reduced intestinal and hepatic microcirculation reflects the combined effects of septic shock and V-A ECMO. Further studies assessing the intestinal microcirculation of healthy rats treated with femoral V-A ECMO are needed to clarify the impact of septic shock.

It has been shown that elevated oxygen concentrations are associated with hyperoxia-induced intestinal injury [28]. Therefore, further studies should include the measurement of reactive oxygen species in the intestine. In addition to mucosa atrophy and enterocyte cell death, leukocyte and macrophage infiltration have been described [29]. Moreover, hyperoxia disrupts the integrity of the intestinal barrier, leading to the translocation of bacteria from the intestine into the bloodstream [30]. Therefore, further analyses focused on cytokine concentrations are needed.

Since septic shock was combined with ECMO-induced inflammation, an increased proinflammatory response was expected in our study. Interestingly, the CXCL2 concentration was only slightly elevated during high-flow V-A ECMO therapy. It must be considered that the rats received LPS from *Escherichia coli*. During septic shock in humans with bacteriemia, the oxygenator membrane is at risk of infection [13,14]. Therefore, our results must be interpreted with caution.

Since the rats treated with V-A ECMO received lung-protective ventilation with reduced respiratory rate and tidal volume, reduced concentrations of proinflammatory markers were expected in the BAL. However, contrary to this expectation, CXCL2 and CXCL5 concentrations were increased only during low-flow V-A ECMO therapy. It must be noted that more blood is pumped through the native lungs during low-flow V-A ECMO therapy than during high-flow V-A ECMO therapy. Our previous studies treating septic shock rats with V-V ECMO found elevated TNF-α, CXCL2, and CXCL5 concentrations in the BAL. Altogether, the combined effects of septic shock, V-A ECMO, and pulmonary blood flow seem to be associated with increased pulmonary inflammation, as evidenced by cytokine concentrations in the BAL. Additional studies using a right ventricular pressure-volume catheter are needed to clarify this issue.

Our study had some limitations. Firstly, while the mucosal microcirculation should be assessed from inside the intestine, the Oxygen To See (O2C) probes were placed outside. Nonetheless, since the probes are designed to measure oxygenation at a depth of 2–4 mm, the results can be used as a surrogate for intestinal microcirculation. Secondly, the findings of animal studies cannot directly be transferred to humans. However, rats have a cardiopulmonary system similar to humans and allow the creation of comparable groups with low variance due to inbreeding. Thirdly, the infusion of LPS from *E. coli* is associated with a cytokine storm rather than bacterial infection, as requested by the Sepsis-3 definition [2]. Another septic shock model is created by ligating and puncturing the coecum to allow bacteria to translocate into the abdominal cavity. However, the onset of septic shock is more heterogeneous than after LPS infusion, resulting in more heterogeneous groups. Additionally, this model is more complex since the animals require a second round of anesthesia and surgery. Moreover, the animals must be monitored between the two procedures, which requires personnel, time, and money. Therefore, regional councils rarely authorize coecum ligature and punction due to animal welfare concerns. Lastly, only rats with lipopolysaccharide-induced septic shock were analyzed. The effect of V-A ECMO on intestinal microcirculation needs to be further investigated in healthy rats.

## 4. Materials and Methods

### 4.1. Animals

All procedures involving animals were conducted in compliance with standards for animal care and the Animal Research: Reporting of In Vivo Experiments (ARRIVE) guidelines and approved by the local committee responsible for animal care (Animal Welfare Commission of the Department of Veterinary Medicine at the Regional Council Giessen—GI 20/26 Nr. G 77/2019; Regierungspraesidium Giessen, Germany).

Male Lewis rats (330–350 g) obtained from Janvier Labs (Le Genest St. Isle, France) were housed at 22 °C, 55% relative humidity, and a 14/10 h day/night cycle, with access to standard chow and water ad libitum. The rats were randomly divided into three groups per lot to receive V-A ECMO therapy with high (V-A EMCO 90) or low (V-A ECMO 60) blood flow or sham procedure (*n* = 10/group). During the sham procedure, all cannulas were inserted and the rats were monitored for two hours without V-A ECMO support.

### 4.2. Induction and Maintenance of Anesthesia

After inhalation induction with 5% Isoflurane (Baxter, Unterschleißheim, Germany) balanced with 95% oxygen, the rats were intubated orotracheally (16 G cannula; B. Braun, Melsungen, Germany) and ventilated with an inspiratory oxygen fraction of 0.5 in a weight-adjusted volume-controlled manner (tidal volume = 6.2 mL × body weight (kg)^1.01^, respiratory rate = 53.3 × body weight (kg)^−0.26^) using a Harvard Inspira ventilator (Harvard Apparatus, Cambridge, UK). The rats were placed on a heating pad, which was adjusted based on the temperature measured by a rectal probe. They were also provided heat with an infrared lamp to regulate the rectal temperature between 36.5 °C and 37 °C.

Then, the electrocardiograph was connected and the lateral tail vein was cannulated percutaneously for the continuous infusion of fentanyl (10 µg/kg/h; Albrecht GmbH, Aulendorf, Germany), midazolam (2 mg/kg/h; Roche, Basel, Switzerland), pancuronium (0.1 mg/kg/h; Inresa, Freiburg, Germany), and a balanced crystalloid solution (5 mL/kg Sterofundin; B.Braun, Melsungen, Germany) [19,23].

### 4.3. Cannulation and Abdominal Laparotomy

The following vascular accesses were placed after surgical preparation. First, the tail artery was cannulated with a 24 G cannula for intermittent blood gas analysis and continuous measurement of the SAP, DAP, and MAP (B.Braun, Melsungen, Germany). Next, after a small skin incision was made in the groin, the femoral artery was dissected and cannulated with a 22 G cannula (Surflo; Terumo, Eschborn, Germany) for arterial inflow from the ECMO circuit.

Following this, the neck was opened with a small incision and the internal jugular vein and carotid artery vein were dissected. Then, a 2 F pressure-volume catheter was carefully moved through the carotid artery into the left ventricle to measure the SV, CO, LVEDV, LVEDP, and LVEF (SPR-838; Millar, Houston, TX, USA).

After a median skin incision was made with scissors, the abdominal cavity was opened via electrocautery (AA01 Bovie high-temperature cautery; Bovie Medical Corporation, Clearwater, FL, USA) as previously described [31]. Next, the intestine was mobilized and the vascular-free mesentery of a small intestine loop was dissected. Then, the probe for the white light and laser Doppler spectrometry (LFX-151; LEA Medizintechnik GmbH, Heuchelheim, Germany) was placed on the intestine and secured with a one-side-open silicon tube (diameter: 8 mm) to assure a loose fit. The whole intestine was put back in its original position. Next, a shallow well was placed on the right lobe of the liver (LFX-45; LEA Medizintechnik GmbH, Heuchelheim, Germany) and the abdominal cavity was covered with a warm, wet compress [31].

Finally, all rats received heparin (400 IU/kg; Merckle GmbH, Blaubeuren, Germany), and the internal jugular vein was cannulated with a modified multi-orifice 17 G cannula (B. Braun, Melsungen, Germany) for venous outflow to the ECMO circuit.

### 4.4. Induction of Septic Shock

Septic shock was induced in all rats by administering 1 mg/kg LPS from *E. coli* O111:B4 (LPS-EB Ultrapure; InvivoGen, San Diego, CA, USA) via a syringe pump (11 Plus; Harvard Apparatus, Holliston, MA, USA) through the draining ECMO cannula over 30 min before commencing ECMO.

### 4.5. Extracorporeal Membrane Oxygenation

As previously described, the ECMO circuit consisted of a venous reservoir (M. Humbs, Valley, Germany), a roller pump (Verderflex Vantage 3000; Castleford, UK), and a membrane oxygenator (Micro-1; Kewei Rising Medical, Shenzhen, China) [19,23]. A Heidelberger extension line (B. Braun, Melsungen, Germany) was wrapped around the oxygenator and connected to a heating pump (HU35; Gettinge, Raststatt, Germany) to prevent heat loss. The whole circuit was primed with 250 IU of heparin (Ratiopharm, Ulm, Germany) and 9 mL of 6% unbalanced hydroxy ethyl starch (Voluven; Fresenius Kabi, Bad Homburg, Germany). The blood flow was started with a rate of 30 mL/kg/min and then continuously increased to the target flow of 60 (V-A ECMO 60) or 90 (V-A ECMO 90) mL/kg/min. The sweep gas flow on the membrane was adjusted between 20 and 50 mL/min to regulate the pCO_2_ between 35 and 45 mmHg. The oxygen fraction on the ECMO membrane was set to 0.5. For lung-protective ventilation, the respiratory rate and tidal volume were set at 75% of the rat’s weight.

### 4.6. Intestinal Microcirculation

To measure the intestinal microcirculation, the intestine and liver probes were connected to the micro-lightguide spectrophotometry O2C device (LEA Medizintechnik GmbH, Heuchelheim, Germany). As described previously, each probe consisted of two light sources and their corresponding optical sensors [31]. White light spectroscopy (450–1000 nm) was used to measure the percentage of SO_2_, which is composed primarily of venous and secondarily of arterial and capillary oxygen saturation. The amount of light absorption due to hemoglobin was analyzed and expressed in relative hemoglobin arbitrary units (RU). The second light source emitted laser light (820 nm, 30 mW) and was used to determine erythrocyte velocity and, thereby, relative blood flow [31].

### 4.7. Timepoints of Hemodynamic Measurements

Baseline values were recorded before administering LPS. The subsequent measurements were captured before commencing ECMO and every 10 min thereafter, up to 120 min.

### 4.8. Blood Analyses

Blood gas analyses were performed immediately before commencing ECMO and then every 30 min thereafter, up to 120 min (ABL800; Radiometer, Copenhagen, Denmark). Recordings included S_a_O_2_, S_cv_O_2_, pO_2_, pCO_2_, hemoglobin, hematocrit, pH, bicarbonate, base excess, lactate, glucose, sodium, potassium, calcium, and chloride. Blood samples were also collected for inflammation analysis immediately after starting ECMO and every 60 min thereafter, up to 120 min. This blood was centrifuged at 5000 rpm for five minutes and the plasma samples were stored at −80 °C until required.

### 4.9. End of Experiments

After 120 min, isoflurane was set to 5% and the rats were euthanized by exsanguination through the draining ECMO cannula. Shortly after confirming death by asystole, the neck was opened and the trachea was dissected. Next, a ligature was fixed around the trachea to seal the tube. Then, the lungs were flushed repeatedly with 40 mL of balanced crystalloid solution (Sterofundin; Fresenius, Bad Homburg, Germany). After centrifuging the BAL at 1200 rpm and 4 °C for eight minutes, the supernatant was collected and stored at −80 °C until needed.

### 4.10. Enzyme-Linked Immune Sorbent Assays

Systemic and pulmonary inflammation was assessed by measuring the concentrations of the cytokines TNFα, IL-6, IL-10, CXCL2, and CXCL5 in the plasma and BAL using enzyme-linked immune sorbent assays R6000B, RTA00, and R1000 from R&D System (Wiesbaden, Germany) and ERCXCL2 and ERCXCL5 from Thermo Fisher Scientific (Waltham, MA, USA) according to the manufacturer’s instructions; the probes were only thawed once.

### 4.11. Statistical Analyses

All data are expressed as the median with the 25th and 75th percentiles. Data were compared between groups using analysis of variance for repeated measures followed by a post hoc Bonferroni test. Since the baseline and first measurements were captured without ECMO support, they were not included in the repeated measures analysis. A *p*-value of <0.05 was considered statistically significant. All statistical analyses were performed using SPSS (version 20; IBM, Stuttgart, Germany). All graphs were created using GraphPad Prism (version 7; GraphPad Software, San Diego, CA, USA).

## 5. Conclusions

Despite increased blood pressure and CO, rats treated with V-A ECMO exhibited impaired microcirculation in the intestine and liver during septic shock. While all rats with septic shock received lung-protective ventilation, increased pulmonary inflammation assessed by BAL was observed during low-flow V-A ECMO therapy. Further studies are needed to clarify the molecular mechanisms of how femoral V-A ECMO therapy-induced lower-body hyperoxia affects the intestine.

## Figures and Tables

**Table 1:** Results of the blood gas analysis.

		0 min	30 min	60 min	90 min	120 min
S_a_O_2_ ***	sham	100 (99–100)	97 (96–98)	95 (94–96)	94 (92–95)	93 (92–95)
**###**	V-A 60	100 (100–100)	100 (100–100)	100 (100–100)	100 (100–100)	100 (100–100)
**(%)**	V-A 90	99 (97–100)	100 (100–100)	100 (100–100)	100 (100–100)	100 (100–100)
**S_cv_O_2_**	sham	80 (77–85)	80 (74–86)	74 (69–79)	74 (70–75)	69 (64–71)
	V-A 60	79 (74–86)	83 (80–89)	80 (78–85)	76 (73–81)	70 (65–76)
**(%)**	V-A 90	78 (73–83)	82 (66–85)	78 (71–82)	69 (61–74)	62 (56–70)
**avDO_2_**	sham	4.4 (3.4–5.2)	3.3 (2.2–4.4)	3.6 (2.9–4.6)	3.3 (2.7–3.9)	3.8 (3.4–4.1)
	V-A 60	4.8 (3.0–5.8)	2.5 (1.8–3.0)	2.8 (2.1–2.9)	3.1 (2.6–3.3)	3.6 (2.9–4.1)
**(mL/dL)**	V-A 90	4.5 (3.7–6.2)	2.9 (2.1–4.4)	3.1 (2.6–3.5)	3.6 (3.3–3.8)	4.3 (3.4–4.9)
pO_2_ ***	sham	130 (115–140)	108 (106–120)	106 (96–110)	98 (90–100)	87 (79 – 94)
**###**	V-A 60	146 (134–158)	202 (192–211)	193 (181–201)	187 (171–196)	178 (153–189)
**(mmHg)**	V-A 90	119 (112–141)	203 (184–238)	188 (168–214)	179 (162–214)	162 (150–196)
pCO_2_ ***	sham	44 (42–46)	58 (52–61)	60 (57–65)	61 (55–66)	60 (55–67)
**###**	V-A 60	40 (39–43)	36 (32–39)	40 (38–42)	38 (37–39)	37 (37–39)
**(mmHg)**	V-A 90	51 (43–55)	37 (35–40)	36 (32–40)	39 (35–42)	37 (35–38)
pH ***	sham	7.37 (7.33–7.39)	7.25 (7.23–7.28)	7.21 (7.19–7.23)	7.23 (7.20–7.26)	7.25 (7.22–7.27)
**###**	V-A 60	7.39 (7.37–7.42)	7.42 (7.39–7.47)	7.38 (7.36–7.40)	7.41 (7.39–7.44)	7.42 (7.41–7.45)
	V-A 90	7.31 (7.29–7.38)	7.41 (7.39–7.44)	7.41 (7.38–7.45)	7.41 (7.34–7.44)	7.42 (7.38–7.46)
BE ***	sham	−1.0 (−2.0–0.7)	−3.9 (−4.8–−2.7)	−4.8 (−6.5–−4.2)	−3.5 (−4.9–−2.0)	−1.8 (−2.9–−1.2)
**###**	V-A 60	−0.3 (−1.0–1.6)	−0.6 (−1.3–0.1)	−1.1 (−2.1–−0.9)	0.0 (−0.5–.3)	1.2 (−0.6–1.9)
	V-A 90	−0.3 (−1.8–0.3)	−0.5 (−2.1–0.3)	−0.9 (−1.7–−0.2)	−0.9 (−1.5–0.1)	−0.5 (−3.0–0.2)
**Lac**	sham	1.3 (1.1–1.5)	1.9 (1.8–2.1)	2.1 (1.8–2.8)	1.6 (1.4–2.2)	1.2 (1.0–1.7)
	V-A 60	1.4 (1.3–1.8)	2.0 (1.8–2.0)	2.2 (2.1–2.3)	2.1 (1.7–2.3)	1.8 (1.4–1.9)
**(mmol/L)**	V-A 90	1.5 (1.1–1.9)	2.1 (1.8–2.3)	2.3 (2.1–2.8)	2.3 (1.9–2.6)	2.1 (1.7–2.7)
Hb ***	sham	15.1 (15.0–15.5)	13.5 (13.2–13.9)	12.8 (12.3–13.3)	11.6 (11.2–11.9)	11.1 (10.3–11.5)
**###**	V-A 60	15.5 (15.1–16.0)	8.3 (8.2–8.7)	8.0 (7.9–8.3)	7.7 (7.5–7.9)	7.3 (7.1–7.9)
**(g/dL)**	V-A 90	15.2 (14.6–15.6)	8.2 (7.7–8.9)	7.8 (7.7–8.2)	7.4 (7.1–7.7)	7.4 (7.1–7.5)
Hct ***	sham	46.4 (46.0–47.4)	41.5 (40.6–42.6)	39.1 (37.9–41.0)	35.8 (34.6–36.6)	34.1 (31.8–35.4)
**###**	V-A 60	47.7 (46.4–48.7)	25.8 (25.4 – 27.1)	24.9 (24.7–25.8)	24.0 (23.5–24.5)	22.7 (22.3–24.5)
**(%)**	V-A 90	46.4 (44.5–47.6)	25.5 (24.0–27.4)	24.4 (23.8–25.5)	23.1 (22.0–23.9)	23.2 (22.1–23.6)
**Glu**	sham	163 (157–171)	213 (181–231)	184 (166–223)	147 (132–160)	138 (120–150)
	V-A 60	180 (168–189)	179 (171–192)	161 (146–168)	138 (126–154)	135 (122–153)
**(mg/dL)**	V-A 90	168 (156–181)	171 (157–182)	157 (151–172)	146 (129–156)	128 (116–140)
**Na**	sham	141 (141–142)	143 (142–143)	143 (143–144)	144 (142–145)	144 (143–144)
	V-A 60	142 (141–142)	143 (142–145)	144 (144–145)	145 (143–145)	145 (144–145)
**(mmol/L)**	V-A 90	142 (141–143)	144 (143–144)	144 (143–145)	145 (144–146)	145 (144–146)
**K**	sham	4.5 (4.5–4.7)	3.9 (3.8–4.2)	3.9 (3.7–3.9)	4.2 ( 4.0–4.4)	4.6 (4.4–4.8)
	V-A 60	4.6 (4.4–4.6)	4.0 (4.0–4.2)	3.9 (3.7–3.9)	4.2 (4.1–4.3)	4.5 (4.3–4.6)
**(mmol/L)**	V-A 90	4.7 (4.4–4.9)	4.3 (4.0–4.4)	4.1 (3.9–4.2)	4.3 (4.1–4.4)	4.7 (4.4–4.8)
Cl ***	sham	109 (108–110)	110 (109–111)	111 (110–112)	111 (111–113)	112 (111–114)
**##**	V-A 60	109 (108–111)	113 (112–114)	115 (114–115)	115 (114–116)	115 (114–116)
**(mmol/L)**	V-A 90	108 (108–110)	113 (112–114)	114 (113–114)	115 (114–116)	115 (115–116)
**Ca**	sham	1.51 (1.50–1.53)	1.47 (1.45–1.49)	1.46 (1.45–1.50)	1.46 (1.40–1.48)	1.43 (1.41–1.44)
	V-A 60	1.48 (1.46–1.53)	1.46 (1.44–1.49)	1.44 (1.41–1.47)	1.41 (1.37–1.42)	1.38 (1.34–1.40)
**(mmol/L)**	V-A 90	1.51 (1.48–1.54)	1.47 (1.46–1.49)	1.42 (1.39–1.42)	1.40 (1.38–1.44)	1.41 (1.38–1.44)

Data are presented as the median with 25th and 75th percentiles. The degree of statistical significance between sham and V-A ECMO 60 is denoted by asterisks (***, *p* < 0.001) and between sham and V-A ECMO 90 by hash marks (^##^, *p* < 0.01; ^###^, *p* < 0.001). Abbreviations: avDO_2_ = arterio-venous oxygen difference; BE = base excess; Bic = bicarbonate; Ca = calcium; Cl = chloride; Glu = glucose; Hb = hemoglobin; Hct = hematocrit; K = potassium; Lac = lactate; Na = sodium; pCO_2_ = arterial partial pressure of carbon dioxide; pO_2_ = arterial partial pressure of oxygen; S_a_O_2_ = arterial oxygen saturation; S_cv_O_2_ = central venous oxygen saturation.

## Data Availability

The raw data supporting the conclusions of this article will be made available by the authors on request.

## Supplementary Materials

The following supporting information can be downloaded at: https://www.mdpi.com/article/10.3390/ijms25137421/s1.

## References

[1] Fleischmann-Struzek C., Mellhammar L., Rose N., Cassini A., Rudd K.E., Schlattmann P., Allegranzi B., Reinhart K. (2020). Incidence and mortality of hospital- and ICU-treated sepsis: Results from an updated and expanded systematic review and meta-analysis. Intensive Care Med..

[2] Singer M., Deutschman C.S., Seymour C.W., Shankar-Hari M., Annane D., Bauer M., Bellomo R., Bernard G.R., Chiche J.-D., Coopersmith C.M. (2016). The Third International Consensus Definitions for Sepsis and Septic Shock (Sepsis-3). JAMA.

[3] Burgdorff A.-M., Bucher M., Schumann J. (2018). Vasoplegia in patients with sepsis and septic shock: Pathways and mechanisms. J. Int. Med. Res..

[4] Evans L., Rhodes A., Alhazzani W., Antonelli M., Coopersmith C.M., French C., Machado F.R., Mcintyre L., Ostermann M., Prescott H.C. (2021). Surviving sepsis campaign: International guidelines for management of sepsis and septic shock 2021. Intensive Care Med..

[5] Boissier F., Aissaoui N. (2022). Septic cardiomyopathy: Diagnosis and management. J. Intensive Med..

[6] Beesley S.J., Weber G., Sarge T., Nikravan S., Grissom C.K., Lanspa M.J., Shahul S., Brown S.M. (2018). Septic Cardiomyopathy. Crit. Care Med..

[7] Peek G.J., Elbourne D., Mugford M., Tiruvoipati R., Wilson A., Allen E., Clemens F., Firmin R., Hardy P., Hibbert C. (2010). Randomised controlled trial and parallel economic evaluation of conventional ventilatory support versus extracorporeal membrane oxygenation for severe adult respiratory failure (CESAR). Health Technol. Assess..

[8] Barbaro R.P., MacLaren G., Boonstra P.S., Combes A., Agerstrand C., Annich G., Diaz R., Fan E., Hryniewicz K., Lorusso R. (2021). Extracorporeal membrane oxygenation for COVID-19: Evolving outcomes from the international Extracorporeal Life Support Organization Registry. Lancet.

[9] Noah M.A., Peek G.J., Finney S.J., Griffiths M.J., Harrison D.A., Grieve R., Sadique M.Z., Sekhon J.S., McAuley D.F., Firmin R.K. (2011). Referral to an extracorporeal membrane oxygenation center and mortality among patients with severe 2009 influenza A(H1N1). JAMA.

[10] Ling R.R., Ramanathan K., Poon W.H., Tan C.S., Brechot N., Brodie D., Combes A., MacLaren G. (2021). Venoarterial extracorporeal membrane oxygenation as mechanical circulatory support in adult septic shock: A systematic review and meta-analysis with individual participant data meta-regression analysis. Crit. Care.

[11] Sato R., Kuriyama A. (2020). Venoarterial Extracorporeal Membranous Oxygenation: Treatment Option for Sepsis-Induced Cardiogenic Shock? A Systematic Review. Crit. Care Med..

[12] Millar J.E., Fanning J.P., McDonald C.I., McAuley D.F., Fraser J.F. (2016). The inflammatory response to extracorporeal membrane oxygenation (ECMO): A review of the pathophysiology. Crit. Care.

[13] Kuehn C., Orszag P., Burgwitz K., Marsch G., Stumpp N., Stiesch M., Haverich A. (2013). Microbial adhesion on membrane oxygenators in patients requiring extracorporeal life support detected by a universal rDNA PCR test. ASAIO J..

[14] Yu Y., Kim Y.H., Cho W.H., Son B.S., Yeo H.J. (2021). Biofilm microbiome in extracorporeal membrane oxygenator catheters. PLoS ONE.

[15] Haussner F., Chakraborty S., Halbgebauer R., Huber-Lang M. (2019). Challenge to the Intestinal Mucosa During Sepsis. Front. Immunol..

[16] Payen D. (2020). The gut as a hidden source of sepsis. Minerva Anestesiol..

[17] Napp L.C., Brehm M., Kühn C., Schäfer A., Bauersachs J. (2015). Heart against veno-arterial ECMO: Competition visualized. Int. J. Cardiol..

[18] Gehron J., Schuster M., Rindler F., Bongert M., Böning A., Krombach G., Fiebich M., Grieshaber P. (2020). Watershed phenomena during extracorporeal life support and their clinical impact: A systematic in vitro investigation. ESC Heart Fail..

[19] Edinger F., Schneck E., Schulte C., Schmidt G., Gehron J., Sander M., Koch C. (2022). Impact of the inspiratory oxygen fraction on the cardiac output during jugulo-femoral venoarterial extracorporeal membrane oxygenation in the rat. BMC Cardiovasc. Disord..

[20] Nahum E., Skippen P.W., Gagnon R.E., Macnab A.J., Skarsgard E.D. (2006). Correlation of near-infrared spectroscopy with perfusion parameters at the hepatic and systemic levels in an endotoxemic shock model. Med. Sci. Monit..

[21] Albuszies G., Radermacher P., Vogt J., Wachter U., Weber S., Schoaff M., Georgieff M., Barth E. (2005). Effect of increased cardiac output on hepatic and intestinal microcirculatory blood flow, oxygenation, and metabolism in hyperdynamic murine septic shock. Crit. Care Med..

[22] Thorén A., Elam M., Ricksten S.E. (2001). Jejunal mucosal perfusion is well maintained during mild hypothermic cardiopulmonary bypass in humans. Anesth. Analg..

[23] Edinger F., Schneck E., Schulte C., Gehron J., Mueller S., Sander M., Koch C. (2020). Comparison of the effect of membrane sizes and fibre arrangements of two membrane oxygenators on the inflammatory response, oxygenation and decarboxylation in a rat model of extracorporeal membrane oxygenation. BMC Cardiovasc. Disord..

[24] Edinger F., Schmitt C., Koch C., McIntosh J.M., Janciauskiene S., Markmann M., Sander M., Padberg W., Grau V. (2021). Application of alpha1-antitrypsin in a rat model of veno-arterial extracorporeal membrane oxygenation. Sci. Rep..

[25] Al-Ani B., ShamsEldeen A.M., Kamar S.S., Haidara M.A., Al-Hashem F., Alshahrani M.Y., Al-Hakami A.M., Kader D.H.A., Maarouf A. (2022). Lipopolysaccharide induces acute lung injury and alveolar haemorrhage in association with the cytokine storm, coagulopathy and AT1R/JAK/STAT augmentation in a rat model that mimics moderate and severe Covid-19 pathology. Clin. Exp. Pharmacol. Physiol..

[26] Riedel G.L., Scholle J.L., Shepherd A.P., Ward W.F. (1983). Effects of hematocrit on oxygenation of the isolated perfused rat liver. Am. J. Physiol..

[27] Thorén A., Nygren A., Houltz E., Ricksten S.-E. (2005). Cardiopulmonary bypass in humans--jejunal mucosal perfusion increases in parallel with well-maintained microvascular hematocrit. Acta Anaesthesiol. Scand..

[28] Dai N., Gu J., Luo Y., Tao Y., Chou Y., He Y., Qin H., Chen T., Fu X., Chen M. (2024). Impact of hyperoxia on the gut during critical illnesses. Crit. Care.

[29] Wang H.-C., Chou H.-C., Chen C.-M. (2023). Molecular Mechanisms of Hyperoxia-Induced Neonatal Intestinal Injury. Int. J. Mol. Sci..

[30] Chen C.-M., Chou H.-C. (2016). Hyperoxia disrupts the intestinal barrier in newborn rats. Exp. Mol. Pathol..

[31] Schmidt G., Pitz L., Markmann M., Schneck E., Sander M., Koch C., Edinger F. (2023). Micro-lightguide spectrophotometry assessment of hepatic and intestinal microcirculation in endotoxemic rats during intravenous treatment with angiotensin II. Eur. J. Pharm. Sci..

