# Supplementary material for: New Insights into Hepatic and Intestinal Microcirculation and Pulmonary Inflammation in a Model of Septic Shock and Veno-Arterial Extracorporeal Membrane Oxygenation in the Rat
Source: Int J Mol Sci. 2024 Jul 6;25(13):7421. doi: 10.3390/ijms25137421 (PMC11242878; doi:10.3390/ijms25137421)
Supplement: Supplementary file 1 [file ijms-25-07421-s001.zip › ijms-3058237-supplementary.pdf]

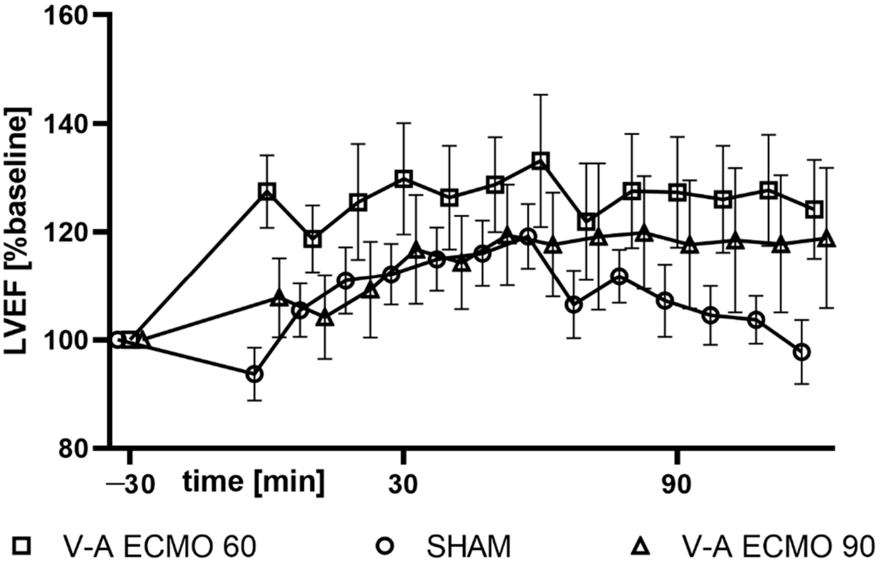

Figure S1. Time course of LVEF. No differences were seen between high and low-flow V-A ECMO therapy compared to sham procedure. Abbreviations: ECMO = extracorporeal membrane oxygenation; LVEF = left ventricular ejection fraction.
